# Separation of H_2_O/CO_2_ Mixtures by MFI Membranes: Experiment and Monte Carlo Study

**DOI:** 10.3390/membranes11060439

**Published:** 2021-06-10

**Authors:** Alexander Wotzka, Majid Namayandeh Jorabchi, Sebastian Wohlrab

**Affiliations:** Leibniz Institute for Catalysis at the University of Rostock, Albert-Einstein-Str. 29a, D-18059 Rostock, Germany; alexander.wotzka@catalysis.de (A.W.); majid.jorabchi@catalysis.de (M.N.J.)

**Keywords:** water, carbon dioxide, ZSM-5, zeolite membrane, Monte Carlo simulation

## Abstract

The separation of CO_2_ from gas streams is a central process to close the carbon cycle. Established amine scrubbing methods often require hot water vapour to desorb the previously stored CO_2_. In this work, the applicability of MFI membranes for H_2_O/CO_2_ separation is principally demonstrated by means of realistic adsorption isotherms computed by configurational-biased Monte Carlo (CBMC) simulations, then parameters such as temperatures, pressures and compositions were identified at which inorganic membranes with high selectivity can separate hot water vapour and thus make it available for recycling. Capillary condensation/adsorption by water in the microporous membranes used drastically reduces the transport and thus the CO_2_ permeance. Thus, separation factors of α_H2O/CO2_ = 6970 could be achieved at 70 °C and 1.8 bar feed pressure. Furthermore, the membranes were tested for stability against typical amines used in gas scrubbing processes. The preferred MFI membrane showed particularly high stability under application conditions.

## 1. Introduction

Amine scrubbing is a chemical absorption process used for natural gas and biogas upgrading [1,2,3,4,5]. Moreover, this technique is considered the most promising method to remove CO_2_ in a post-combustion process. Compared to physical methods, amine scrubbing offers the highest product purity and higher loading rates. 

In the first step of amine washing, absorption takes place in countercurrent, loading the solvent with CO_2_. Currently, mainly monoethanolamine (MEA), diethanolamine (DEA), and methyldiethanolamine (MDEA) are used as detergents [3]. The second step is the desorption of the CO_2_ from the solution. For this, the loaded CO_2_ is mainly desorbed at ~120 °C from the solution [6]. Water and carbon dioxide are discharged as hot waste gases. However, it is recommended that desorption be carried out at the lowest possible temperatures (e.g., 100 °C) [4] in order to keep decomposition and energy costs low. In addition, there are always new developments that show that it is already possible to desorb CO_2_ at significantly lower temperatures [7].

Membranes can be an alternative or useful addition to existing methods to make established processes more energy efficient. Silica membranes are used, for example, for gas separation [8,9,10]. Among others, the separation of H_2_/CO_2_ or the separation of CO_2_ from CH_4_ [8,9] is described. For flue gas treatment, CO_2_/N_2_ separation [10] is known. Other inorganic membranes are zeolite membranes, such as that of the MFI type. Using MFI membranes, the separation of CO_2_ from process gases has already been described in the literature [11]. Often a preferential permeation takes place, which is due to the preferential adsorption on the membrane surface. For example, a selectivity of CO_2_ toward CH_4_ [11] was found. This effect is also used to separate CO_2_ from gas streams. Interestingly, a reduction of CO_2_ permeances has been described with the addition of small amounts of water (1–3%) [12]. Direct separation of CH_4_/CO_2_ on zeolites as an alternative to amine scrubbing is also discussed, but water also interferes with this separation process [13]. Junaidi et al. have also pointed out that CO_2_ permeance is reduced in a wet mixture compared to a dry gas [14]. Here, too, the adsorption principle takes effect. In this case, however, it is the water that adsorbs preferentially that causes this effect. We, too, have already made use of this principle in previous work by using a water-selective membrane to intensify the process of a catalytic reaction [15] or, more distantly, for separating condensable hydrocarbons from natural gas mixtures [16]. 

Membrane processes specifically for water recovery were already reviewed by Bolto et al. [17], wherein the authors discussed several applications such as (i) dehydration of natural gas; (ii) drying of compressed air; (iii) flue gas dehydration; (iv) dehydration of ethanol; (v) dehydration of isopropanol; (vi) dehydration of acetonitrile; but also (vii) steam recovery. Most of the membrane materials described are polymer-based. Only a few inorganic membranes have been reported for these purposes so far and most of them were applied for alcohol dehydratisation [18]. However, the potential of robust inorganic materials for water separation from gas streams has been increasingly recognized in recent times, so that there is now increased research activity in the field. In this way, porous hollow composite membranes were applied for the water separation from flue gas [19], membrane condensers were applied to make use of clean water from power plants [20], or ceramic membrane tubes were used to recover moisture and heat [21], just to mention a few variants of processing. In 2021, Kim and Drioli reviewed state-of-the-art of membrane-based dehydration concepts [22] and they subdivided the available technologies into (i) water separation membranes; (ii) conventional membrane condensers, and (iii) transport membrane condensers. For the first type, only applications like drying of natural gas [23] and compressed air [24] were described. The application potential of zeolite membranes was almost not visible. This was also evident in a second research paper. Again in 2021, a review was published on contemporary CO_2_ separation with zeolites and derived materials as adsorbents or membranes [25]. Here, too, no weight was given to water separation from CO_2_. Consequently, with this contribution we want to extend the present available approaches by including the application of microporous membranes for water separation from carbon dioxide, especially against the background of making such separation techniques available for CO_2_ capture.

## 2. Materials and Methods

Molecular simulations were employed to analyse the performance of MFI zeolite. To simulate the isotherms of pure components and their mixtures, configurational-biased Monte Carlo (CBMC) simulations were conducted using the RASPA software package [26]. All simulations were carried out in the grand canonical ensemble, where temperature T, volume V, and chemical potential µ of the adsorbed species were fixed. To have a chemical equilibration, it is necessary to exchange particles with the reservoir successfully. A more detailed description of the CBMC can be found in [27]. Our simulations consisted of 2.5 × 10^5^ cycles in which the first 2 × 10^4^ were initialization cycles. Periodic boundary conditions were applied to a simulation box consisting of 2 × 2 × 2-unit cells of the MFI-type zeolite. Lorentz–Berthelot mixing rules were employed for interaction between different atom types. To consider the long-range electrostatic interactions, the Ewald summation [28] with a relative precision of 10^−6^ was used.

Force field parameters of the adsorbed specious CO_2_ and H_2_O are shown in the Table 1. The CO_2_ model [29] used is a simple site-based intermolecular potential model that uses point charge and Lennard-Jones interactions centered at each atom. It is a EPM2 model, which is a rescaling model of EPM and can predict critical properties quite well and very close to experimental data. The model also can reproduce the liquid-vapor coexistence properties very accurately.

For H_2_O we used the TIP5P-E model [30], which is a five-site transferable interaction potential model. It is a modified version of the TIP5P model [31] and reproduced experimental data pretty well and predicted all properties of water with high accuracy. The TIP5P-E model shows a density maximum of water near 4 °C and reproduces the thermodynamic, dielectric, and dynamical properties of liquid water over a wide range of temperature and densities. Both models have been used by many scientists as well as by us in our recent paper [32] for a variety of chemical systems and could reproduce both dynamic and thermodynamic properties very well.

MFI membranes were manufactured following an established synthesis procedure [16]. Asymmetric porous corundum tubes (Fraunhofer Institute for Ceramic Technologies and Systems (IKTS), Hermsdorf, Germany) served as membrane supports. The tubes with single-channel geometry (l = 12.5 cm, inside = 0.7 cm, outside = 1.0 cm) were coated on the inside. 

The following steps were carried out in detail: First, the corundum tubes were thermally treated at 130 °C for 12 h. The subsequent seed functionalisation was carried out with suspension consisting of (i) 0.92 g hydroxypropyl cellulose, 8.75 g ethanol, and 8.75 g water and (ii) silicalite particles (5% zeolite solid, type TZP 9023) at room temperature via impregnation. Subsequently, the tubes prepared in this way were dried and calcined at 700 °C for 1 h. The actual membrane synthesis was then initiated via a hydrothermal synthesis. The hydrothermal solution contained 35.64 g tetrapropylammonium hydroxide (TPAOH), 9.34 g tetrapropylammonium bromide (TPABr), and 1.24 g sodium hydroxide (NaOH), which were each dissolved in 50 g of water then mixed under stirring conditions with an additional 50 g of water for at least 5 min. Afterward, 221.2 g of Levasil was added dropwise at room temperature under stirring conditions. The stirring was continued for half an hour and then the solution was left without stirring for 90 min. After ageing of the synthesis solution, the seed-functionalised tubes along with this synthesis solution were transferred to a stainless-steel autoclave with a Teflon top and the membrane growth was carried out for 48 h at 180 °C under autogenous pressure. The zeolite membranes were washed and dried. The subsequent calcination was carried out at 450 °C in air for 6 h.

A commercially available asymmetric silica membrane from the Fraunhofer Institute for Ceramic Technologies and Systems (IKTS) (Hermsdorf, Germany) was used for comparison with the MFI membranes. The silica membrane (1.0 nm pore size) was also a tubular membrane with 1-channel geometry with the dimensions l = 12.5 cm, inside = 0.7 cm, outside = 1.0 cm, and a membrane area of 22.0 cm. The two end faces were also glazed by the manufacturer to 1.5 cm at both ends to prevent leakage.

**Figure 1:**
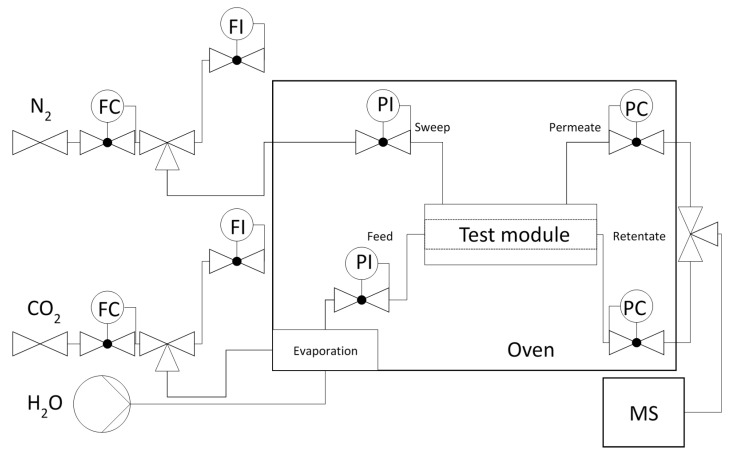
Schematic representation of the experimental set-up used for the separation H_2_O/CO_2_. FC, flow controller; FI, flow indicator; PI, pressure indicator; PC, pressure controller; MS, mass spectrometer.

The membranes were tested in a temperature-controlled test module. The test set-up corresponded with Figure 1 and allowed for the regulation and control of feed and sweep flow, feed and permeate pressure, feed composition, and temperature from dosing to sampling. 

The gases used were nitrogen as the sweep gas (purity 5.0 (9.9999%)) and CO_2_ as the feed gas (Linde purity 4.5 (99.995%)). Feed and sweep flows of 3–21 l/h were used for the experiments. Dosing was done with mass flow controllers (designed for a total flow of 1000 mL/min, MKS Instruments, Andover, USA). Back-pressure valves were used to set the required feed and permeate pressures. The membrane module was tempered in an oven during the tests and the H_2_O evaporator was operated at 120 °C to ensure complete evaporation. To protect against condensation, all lines from the evaporator to the mass spectrometer were heated constantly at 130 °C. The volumetric flow rates of feed and sweep were controlled by means of ADM flow meters (ADM3000, Agilent Technologies, Santa Clara, CA, USA) at the beginning of the experiments. During the experiments, the permeate and retentate flows were also controlled using flowmeters. An ADM3000 from Agilent Technologies (Santa Clara, USA) was used for low water volumes and an Optiflow570 from Carl Stuart Limited (Dublin, Ireland) for high water volumes. The composition of the permeate and retentate was analysed online with a GSD 320 O2C quadrupole mass spectrometer OmniStar, Pfeiffer Vaccum (Asslar, Germany). Two yttrated iridium filaments, together with a secondary electron multiplier C-SEM (detection limit 1 ppm) and Faraday detector (detection limit 40 ppm), were used as detectors; the possible mass range of the instrument used is in the range of 1–200 amu. 

Scanning electron microscopy (SEM) was performed using a JSM-6700F (JEOL, Akishima, Japan) and XRD measurements were performed on an X’Pert PRO reflexion diffractometer from PANalytical (Almelo, The Netherlands). The measurements for determining permporosimetry were carried out with nitrogen as the inert gas and n-hexane as the condensable component. For the measurements, the heated membrane was installed in the membrane module. The measurements were carried out at room temperature and at a pressure difference of 1 bar (p_Feed_ = 2 bar; p_Permeate_ = 1 bar). The n-hexane was tempered to 56 °C in the saturator. The dosage of nitrogen was controlled by a mass flow controller (MKS Instruments, Santa Clara, USA). The measurements could be carried out at partial to saturation vapour pressure ratios (p/p_sat_) of 0.01 to 0.9.

The permeance of component i, Π_i_ as a measure of the selective separation of the membrane was used as an evaluation parameter, see Equation (1) [33],

(1) $$\Pi_{i}=\frac{c_{i,Perm}\cdot {\dot{V}}_{Perm}}{A_{M}\cdot \left(c_{i,Perm}\cdot p_{Feed}-c_{i,Perm}\cdot p_{Perm}\right)}$$

$\Pi_{i}$ is the permeance of compound *i*, *c* the concentration, ${\dot{V}}_{Perm}$ the volume flux in permeate, $A_{M}$ the membrane area, p_feed_ the feed pressure, and p_perm_ the permeate pressure.

Moreover, the separation factor $\alpha_{c_{i}/c_{j}}$ was used as an evaluation parameter, which represents a comparison of the mass ratio in permeate and retentate, see Equation (2).

(2) $$\alpha_{c_{i}/c_{j}}=\frac{{\left[c_{i}/c_{j}\right]}_{Perm}}{{\left[c_{i}/c_{j}\right]}_{Ret}}$$

## 3. Results

### 3.1. Membrane Stability in the Presence of Amines

**Figure 2:**
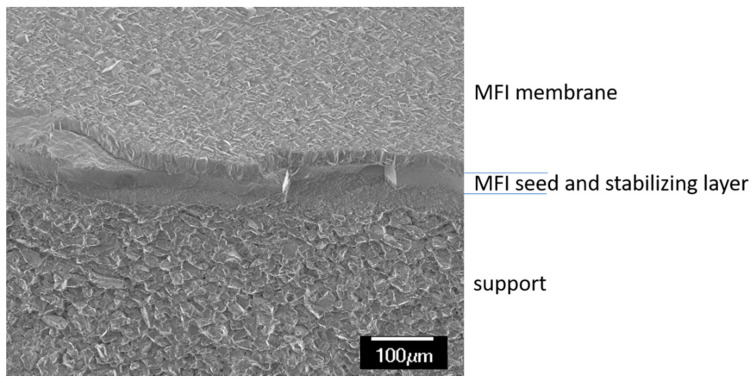
SEM micrograph of a fracture of a fresh MFI membrane.

First, the chemical resistance of the MFI material under separation conditions was established. Thus, experiments were necessary to indicate the stability of MFI membranes in the presence of amines, such as may occur from scrubbing solutions in the stripping gas. For this purpose, a solution consisting of 2.97 g/L piperazine and 5.15 g/L diethanolamine was prepared and an MFI membrane was exposed to this basic solution for 7 days. A supported MFI separating layer (thickness = 42 μm) was used as the membrane, which was additionally mechanically stabilised by a porous MFI intermediate layer [16]. A microporous SiO_2_ separation layer (d_pore_ ~ 1 nm) served as a reference membrane. Figure 2 shows an SEM image of a fresh MFI membrane.

**Figure 3:**
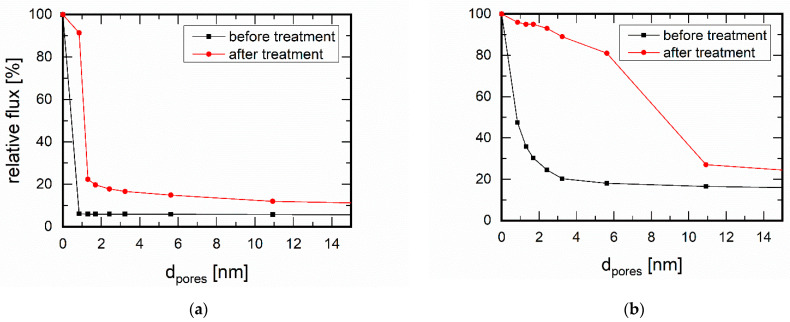
Permporometry measurements on (**a**) an MFI membrane and (**b**) a silica membrane (d_pore_ = 1 nm) before and after amine treatment (2.97 g/L piperazine and 5.15 g/L diethanolamine for 7 days), measured with N_2_ as the inert gas and n-hexane as the condensable component.

**Figure 4:**
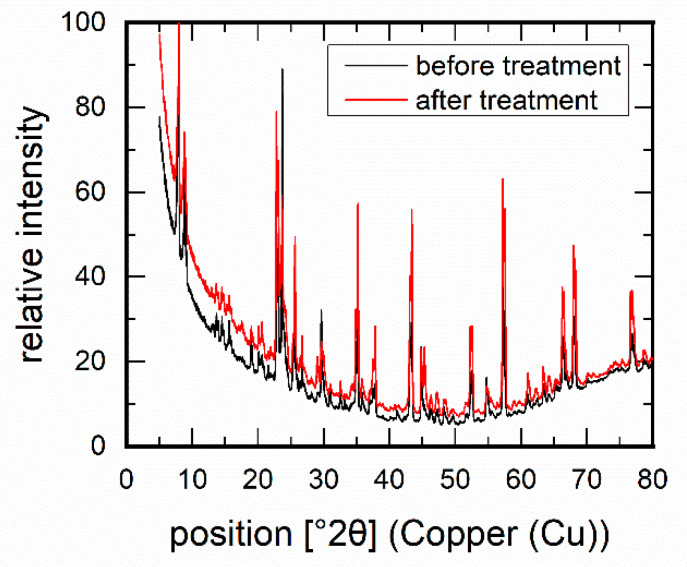
XRD measurements on an MFI membrane before and after amine treatment (2.97 g/L piperazine and 5.15 g/L diethanolamine for 7 days).

The characterisation of the membranes via permporometry [34,35] provided information on any changes that may have occurred in the membrane layer during this treatment. Permporometry uses the adsorption behaviour of an easily condensable component, in this case n-hexane, to close the membrane pores for gas permeation, in this case N_2_. Via the partial pressure of the condensable component, changes in pore size, number, and type of defects as well as pore closure can be determined. The zeolite structure used in this work belongs to the class of MFI structures (Mobile Five). MFI is characterised by its channels with 10-membered rings in the crystallographic b-direction [36]. These open pores have a size of 0.53 nm × 0.56 nm. Furthermore, elliptical pores run along the crystallographic a-axis and have dimensions of 0.51 nm × 0.57 nm. In these pores, substance-selective adsorption of gas molecules can take place. The ratio p/p_s_ corresponds to the quotient of the partial pressure of the condensable component to its saturation pressure p_s_. This ratio was correlated to a pore size up to which the blocking of the membrane for the non-condensable gas occurs. MFI membranes showed before amine treatment that a majority of the pores were below 0.85 nm (Figure 3a), which is true for the pore system of the MFI structure. After treatment, however, larger pores were also found to a certain extent. These pores were most likely formed via attacking of the edges between the crystals, thus forming more voids or enlarging existing voids. However, the structural integrity was proven by XRD, where small changes in reflection intensities were likely to be due to textural differences. (Figure 4).

The silica membrane showed an even more distinct change after amine treatment (cf. Figure 3b). The pores of this membrane were up to 3.23 nm in size before treatment. The pore sizes here were not defined as with a zeolite structure but showed a distribution. In addition, a high leakage flux already existed with this membrane. After amine treatment, the pores became much larger. From the permporometry measurements it is clear that they were widened to up to 10.9 nm. In addition, the leakage flux increased.

In the comparison of both membranes, significantly lower effects of the amine components on the MFI membrane were observed. Obviously, the crystalline MFI structure had a higher stability in basic solution than the microporous SiO_2_. For further investigations, we had now first investigated the separation of water from CO_2_ gas streams, as shown below. Due to the absence of a liquid phase, no mass transport in the form of leaching could take place, which justified this procedure. Nevertheless, the MFI membrane treated with amine solution was examined by a final separation experiment, which even showed an improvement in performance, as will be shown in the conclusions.

### 3.2. Determination of Possible Separation Conditions via Monte Carlo Simulation

**Figure 5:**
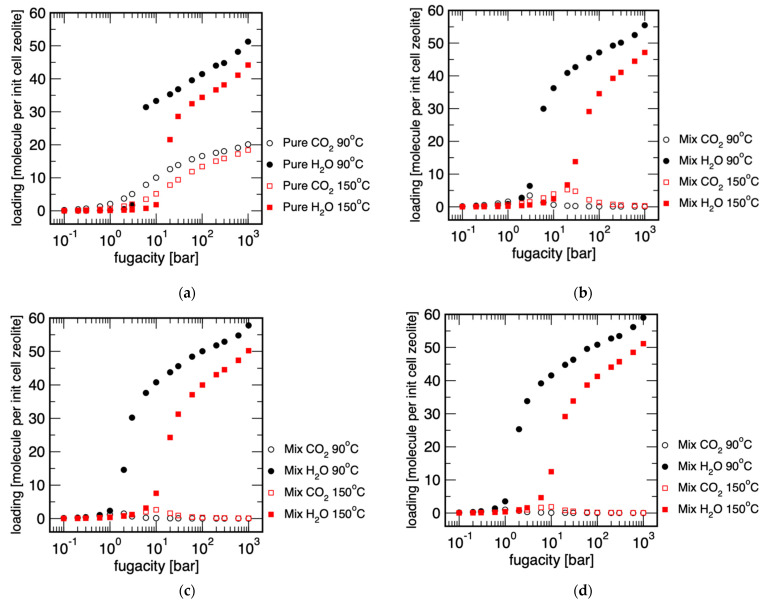
Adsorption isotherms of (**a**) pure and H_2_O/CO_2_ mixture ratios of (**b**) 0.3, (**c**) 0.7, and (**d**) 1.0 in an MFI unit cell at 90 °C and 150 °C calculated from grand canonical Monte Carlo simulations.

Monte Carlo simulations were carried out to narrow down the parameter space of an adsorption-mediated selective membrane separation. With these calculations, a significantly wider parameter space can be achieved than in comparison to experimental measurements on powders. Let us first look at the adsorption of the individual gases in the MFI zeolite (Figure 5). Both at 150 °C and 90 °C, above a certain pressure, significantly more water molecules were adsorbed than carbon dioxide molecules. It is interesting that the adsorption of water at a temperature of 90 °C increased abruptly in the range of 2–4 bar and at 150 °C between 10 and 11 bar. The adsorption behaviour of carbon dioxide tended to increase steadily with increasing pressure. This behaviour changed significantly when the two molecules were mixed. We have already observed the principles of the behaviour described below in the separation of condensable n-butane from methane [37]. 

To the best of our knowledge, there is no previous research on theoretical data of this zeolite type for H_2_O/CO_2_ adsorption and studies have been limited to the pure gas adsorption. Desbiens et al. [38] reported the grand canonical Monte Carlo simulations of the gas and liquid phase adsorption of H_2_O in silicalite-1 zeolite and found this method and MFI zeolite effective for studying gas adsorption in nanoporous materials. Ektefa et al. [39] reported the performance of some all-silica zeolite, including BEA (beta), FAU (faujasite), MFI (silicalite-1), and MOR (mordenite), to adsorb H_2_O using grand canonical Monte Carlo simulations and figured out that the MFI is comparable with other zeolites and has great performance in terms of H_2_O adsorption. Adsorption isotherms of an ethanol/water system were calculated by Lu et al. [40] using MFI and other zeolites as well as carbon nanotubes, and they revealed that MFI zeolites are great candidates for water separation.

Figure 5 shows the adsorption isotherms of water, CO_2_, and the mixtures of H_2_O/CO_2_ with ratios of 0.3, 0.7, and 1.0, respectively, in the MFI structure, more precisely in an MFI unit cell. The chosen conditions represent water-lean and water-rich off-gases from stripping processes. In addition, two temperatures were chosen: 150 °C, which corresponded with classical amine scrubbing with high-temperature desorption, and 90 °C, which represented the temperature condition of a low-temperature treatment, as these have been increasingly investigated in recent years. The pressure dependence of the adsorption is important here. It specifies the range of validity of the separation of substances on MFI membranes: these theoretical considerations show the application range of MFI membranes in H_2_O/CO_2_ separation. Generally, it can be seen that an increase in pressure increased the adsorption of water both as a single component and in a mixture with CO_2_ on the membrane surface. In detail, one can see an influence of the H_2_O/CO_2_ ratio. At 90 °C, the water from a mixture with a H_2_O/CO_2_ ratio of 0.7 condensed from about 2 bar in the MFI structure, whereas at a mixture with a lower H_2_O/CO_2_ ratio of 0.3, about 6–7 bar were necessary for pore blocking with water. This means that above these pressures the membrane became selective for water and this knowledge is important if the possible working range of the membrane is to be adapted to the off-gas of the stripping process. Similarly, one can deduce the influence of temperature on adsorption and thus membrane performance: at higher temperatures, higher pressures become necessary for adsorption, leading to pore blocking and thus separation selectivity. In the case of the mixture with the H_2_O/CO_2_ ratio of 0.7, the process pressure required for separation thus increased from 2 to over 10 bar when the process temperature was increased from 90 °C to 150 °C.

With these theoretical considerations, we have now conceptualized the separation experiments described below. In the next subsection, we will report on the practically determined influence of the temperature, the pressure and the amount-of-substance fraction on the separation process.

### 3.3. Separation of CO_2_/H_2_O Gas Streams at MFI Membranes

#### 3.3.1. Influence of Temperature

One of the most significant factors influencing permeability and thus separation is the process temperature [41]. At correspondingly low temperatures, surface diffusion usually takes place in zeolite membranes, which is enhanced for water by the hydrophilicity of the MFI zeolites. When the temperature is increased, this effect is reduced, but the high temperatures activate gas diffusion at the same time [42]. 

**Figure 6:**
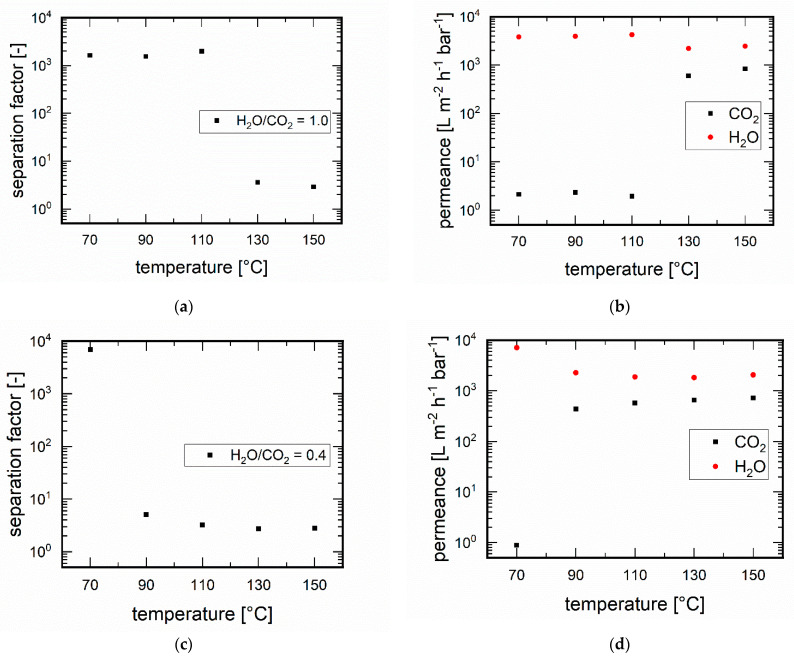
Temperature dependence of the separation at an H_2_O/CO_2_ ratio of 1.0 the (**a**) separation factors and (**b**) permeances, and at an H_2_O/CO_2_ ratio of 0.4 the (**c**) separation factors and (**d**) permeances.

First, we investigated water-rich H_2_O/CO_2_ mixtures and started with a ratio of 1.0 (Figure 6a). At temperatures from 70 °C to 110 °C, surprisingly good separation factors were found between values of 1540 and 1983, with an increasing tendency with a temperature increase from 70 to 110 °C, regardless of whether the mixture was above (70 °C) or below (90 °C and above) the saturation vapour pressure curve for water. This can be explained by the increasing mobility of the adsorbed molecules. However, the separation efficiency dropped sharply above 110 °C. Obviously, above this temperature a complete pore blocking was no longer guaranteed as also implied by the Monte Carlo simulations from the previous section. The H_2_O permeances showed a similar course (see Figure 6b). At temperatures up to 110 °C, the H_2_O permeance increased, favoured by the higher diffusion, from 3694 Lm^−2^h^−1^bar^−1^ at 70 °C to 4245 Lm^−2^h^−1^bar^−1^ at 110 °C. At 130 °C it dropped by a factor of two (2203 Lm^−2^h^−1^bar^−1^) compared to the lower temperature. This decline, however, does not explain the loss in selectivity. It was due to the CO_2_ permeance, which was almost constantly low up to 110 °C (1.96 Lm^−2^h^−1^bar^−1^) but increased tremendously at 130 °C to 599 Lm^−2^h^−1^bar^1^ and increased further at 150 °C to 839 Lm^−2^h^−1^bar^−1^. CO_2_ nearly not permeates at temperatures up to 110 °C, which explains the high separation factor. With a further increase in temperature, mainly the strong increase in CO_2_ permeance through the opened MFI pores explains the significantly reduced separation factor.

With a reduced water content, specifically with an H_2_O/CO_2_ ratio of 0.7, the separation behaviour should also change. As already suggested by the Monte Carlo simulations, a lowering of the water content was expected to lead to a lowering of the maximum temperature at which pore blocking by water occurs, which is essential for the separation process. This was confirmed experimentally (Figure 6c,d). The separation factor at a temperature of 70 °C was still at a high value of 6970 and the H_2_O permeance at 7096 L⋅m^−2^⋅h^−1^⋅bar^−1^. Moreover, the CO_2_ permeance was very low at 0.9 L⋅m^−2^⋅h^−1^⋅bar^−1^. But already at a temperature of 90 °C, the CO_2_ permeated much more strongly with 436.4 L⋅m^−2^⋅h^−1^⋅bar^−1^. With a further temperature increase to 150 °C, the CO_2_ permeance increased further to 723 L⋅m^−2^⋅h^−1^⋅bar^−1^. The separation factors dropped drastically in the temperature range to 2.7 and 5.1 and the H_2_O permeance was also much smaller (1825–2265 L⋅m^−2^⋅h^−1^⋅bar^−1^) due to the competitive adsorption/diffusion with CO_2_.

#### 3.3.2. Influence of Pressure

**Figure 7:**
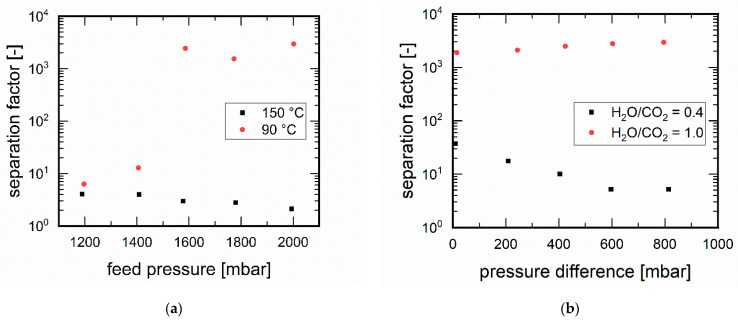
Dependence of pressure: (**a**) feed pressure-dependent separation factors at temperatures of 150 and 90 °C, at feed pressures of 1.0 to 2.0 bar; permeate pressure 1.0 bar, H_2_O/CO_2_ ratio = 1.0; (**b**) permeate pressure-dependent separation factors for the H_2_O/CO_2_ ratios of 0.4 and 1.0, retentate pressure at 1.8 bar.

The influence of the pressure on the separation was investigated. Figure 7a shows for the temperatures of 90 °C and 150 °C the dependence of the separation on the feed pressure at a constant permeate pressure of 1.0 bar. The H_2_O/CO_2_ ratio was 1:1. At 90 °C, insufficient separation was shown at low feed pressures up to 1.4 bar. At higher feed pressures, however, the separation factor achieved rises to well over 1000—the cause here was also the onset of pore blocking due to water adsorption. At 150 °C the separation factors were at a low level over the entire measuring range and tended to decrease with increasing pressure difference. Under these process conditions, no water was adsorbed to the extent that the membrane became selective toward carbon dioxide.

Furthermore, the permeate pressure was varied at a constant feed pressure of 1.8 bar. In addition, an interesting behaviour was observed as a function of the H_2_O/CO_2_ ratio (cf. Figure 7b). As the permeate pressure increased, i.e., the pressure difference decreased, the CO_2_ permeance decreased at an H_2_O/CO_2_ ratio of 0.4. At a permeate pressure of 1.0 bar, i.e., a pressure difference of 0.8 bar, the CO_2_ permeance was 377.0 L⋅m^−2^⋅h^−1^⋅bar^−1^, whereas at a permeate pressure of 1.8 bar (pressure difference 0.0 bar) it decreased to 49.9 L⋅m^−2^⋅h^−1^⋅bar^−1^. The influence of applied pressure differences was in accordance to previously described performance of MFI membranes in the separation of alkanes [37] and followed an increased permeation flux induced by a higher pressure difference between permeate and feed [43,44]. Looking at the decreasing CO_2_ permeances at increasing permeate pressure, i.e., the pressure difference decreased, the increase in separation factor for the H_2_O/CO_2_ ratio of 0.4 can be explained. At the H_2_O/CO_2_ ratio of 1.0 the CO_2_ permeance was constant at a low level between 1.0 and 1.3 L⋅m^−2^⋅h^−1^⋅bar^−1^, which resulted in high separation factors for the H_2_O/CO_2_ ratio of 1.0. A lower permeate pressure increased the desorption rate of H_2_O and increased the separation factor further, which explains the increase in separation factor for the H_2_O/CO_2_ ratio of 1.0 with increasing pressure difference.

#### 3.3.3. Influence of Feed Flux

**Figure 8:**
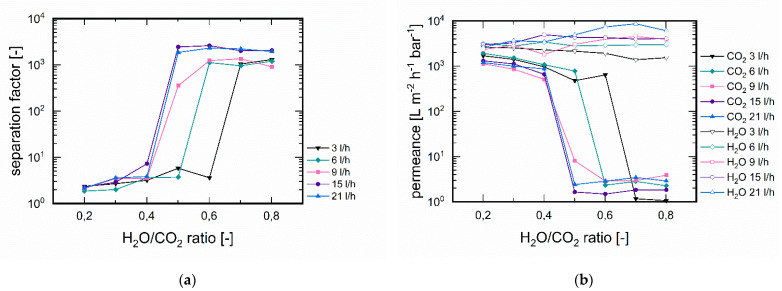
Influence of feed flux on (**a**) separation factors and (**b**) H_2_O and CO_2_ permeances. The sweep flow was adjusted to the feed flow and is the same in each case (T = 90 °C, p_feed_ = 1.9 bar, p_permeate_ = 1.0 bar).

The different separation efficiencies were due to the separation principle. At low H_2_O/CO_2_ ratios, water adsorbed preferentially on the pore wall and was transported across the membrane via surface diffusion only with satisfactory selectivity (Figure 8a). However, above a certain concentration of water, an adsorption point was reached, which almost completely blocked the pores for CO_2_ but did not reduce the permeation of H_2_O. This behaviour could also be seen from the achievable permeances in Figure 8b. Interestingly, by increasing the flow of the gases with smaller proportions of water, highly efficient separation could also be achieved. This was due to sufficient saturation of the adsorbent component water along the membrane. With small fluxes, the membrane could not be completely saturated with water so that a breakthrough of CO_2_ occurred. The corresponding minimum flows of the respective H_2_O/CO_2_ ratios could be read directly from Figure 8a from the increase in the separation factor. 

#### 3.3.4. Separation Performance of Amine-Treated MFI Membranes

Before and after the amine treatment described in 3.1, the separation behaviour of the MFI membranes was investigated (Table 2). Here it can be seen that the separation factors of the MFI membrane before treatment increased with increasing H_2_O/CO_2_ ratio. After amine treatment, the separation factor increased significantly at an H_2_O/CO_2_ ratio of 0.6 but did not increase to the same extent at a higher H_2_O/CO_2_ ratio of 1.0. A comparison of the permeances showed a similar effect here. The CO_2_ permeance decreased significantly after amine treatment at an H_2_O/CO_2_ ratio of 0.4. However, this did not lead to a deterioration of the separation factor since the H_2_O permeance also decreased. Toward the high H_2_O/CO_2_ ratio, the CO_2_ permeance rose to a higher level than before the amine treatment, and the H_2_O permeance was also higher here than in the comparison measurement. This course, like the permporometry measurements, indicates that the membranes are attacked by the basic amines. However, separation was still possible; in an average range around the H_2_O/CO_2_ ratio of 0.6, the separation factor even increased, so it can be assumed that the MFI membrane can be used for the application.

## 4. Discussion

**Figure 9:**
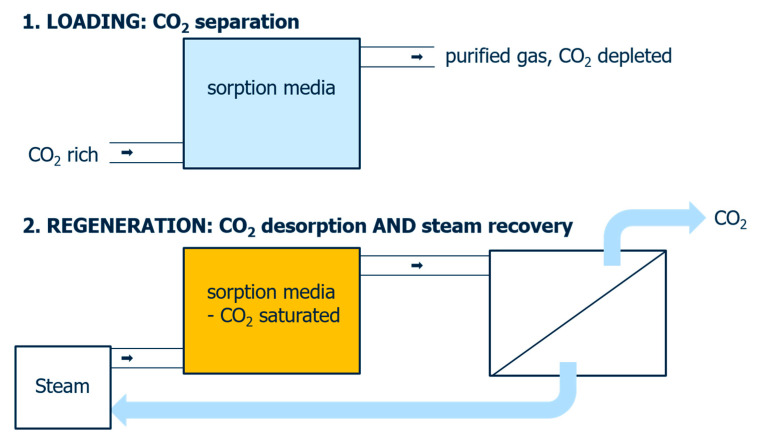
Two-step CO_2_ enrichment process including (1) loading of a sorbent with CO_2_ and (2) regeneration via steam with membrane separation downstream and an integrated water/heat recycle.

Water and heat removal from flue gas by means of porous membrane contactors is adequately described [45]. With the help of membranes, the focus in the past was also on the separation of CO_2_ by permeation [46]. In order to classify the present work, one must recall the underlying separation principle once again. Gaseous water and carbon dioxide are present in different mixtures and are adsorbed to different degrees on the MFI structure. This is to the great advantage of water, which is also preferentially permeated and enriched in the permeate. As mentioned in the introduction, media containing amines are the means of choice for CO_2_ separation from gas streams. The energy needed to desorb the adsorbed CO_2_ must be supplied from outside. We would therefore like to mention here what the possibility of an H_2_O/CO_2_ separation as described here would offer. However, the temperature conditions under which classical amine washes operate are often too high and the separation described herein would be insufficient. Therefore, we address the described separation to novel methods for the regeneration of sorbents that have been preloaded with CO_2_. Such a steam stripping makes it possible to apply heat to materials or media loaded with CO_2_ in a targeted manner [47]. The water introduced as steam initiates the CO_2_ desorption process and an energetically favourable subsequent separation of the H_2_O/CO_2_ mixture that is now produced must be found. The way of separating water and heat via MFI membranes presented in this paper constitutes this possibility. Figure 9 outlines the principle. In Figure 9 (1) first a medium is loaded with CO_2_ and a CO_2_-depleted gas is obtained. In a second step (Figure 9 (2)), CO_2_ is desorbed from the loaded medium by steam, and, in a downstream stage, water is separated from carbon dioxide, whereby the former can be fed back into the process as steam in an energy-efficient manner.

Finally, we would like to present the method of separating H_2_O/CO_2_ mixtures on MFI membranes also as an energy-efficient method for desorption of previously adsorbed CO_2_ from the air. Intensive material developments are currently underway in this regard [48,49,50,51,52,53,54] and in many cases steam-mediated desorption would be worth testing. In recent years, work on CO_2_ harvesting from the atmosphere has intensified, and especially for such high-volume applications, the possibility of energy-friendly process components is essential for future realization.

## 5. Conclusions

In general, depending on the H_2_O/CO_2_ ratio, the separation via MFI membranes of such mixtures is influenced. At lower H_2_O/CO_2_ ratios, a poor separation performance with separation factors below 10 was observed. With a higher H_2_O/CO_2_ ratio, a significantly higher separation level was achieved so that excellent separation factors of over 1000 could be reached. Moreover, with increasing feed flow, this effect already occurs at lower H_2_O/CO_2_ ratios, which is important for possible applications. 

In this work we used low-Al MFI zeolite membranes. However, zeolites as aluminosilicates have a different number of acid sites—which also vary in their acidity—depending on the Si/Al ratio. It is of great interest to address this property in further membrane development in order to gain knowledge about the influence of this parameter on the separation of H_2_O/CO_2_ mixtures.

## Figures and Tables

**Table 1:** Force field parameters of CO_2_ [29] and H_2_O [30] molecules.

	*m*/u	*q*/e	$\varepsilon \cdot k_{B}^{-1}/K$	𝞼/Å
O_CO_2_	15.9994	−0.3256	80.507	3.033
C_CO_2_	12.0	0.6512	28.129	2.76
O_H_2_O	15.9994	0.0	89.633	3.097
H_H_2_O	1.008	0.241	-	-
V_H_2_O	0.0	−0.241	-	-

**Table 2:** Separation behaviour of the MFI membranes before and after the amine treatment.

H_2_O/CO_2_ Ratio	Change of the Separation Factor (–)	CO_2_ Permeance (L⋅m^−2^⋅h^−1^⋅bar^−1^)	H_2_O Permeance (m^−2^⋅h^−1^⋅bar^−1^)
0.4	2.37	6936	12893
	↓	↓	↓
	3.43	2630	7981
0.6	217	26.5	6604
	↓	↓	↓
	401	16.4	7417
1.0	726	9.0	7429
	↓	↓	↓
	505	14.8	8584
